# The Impact of Drying Methods on the Quality of Blanched Yellow Mealworm (*Tenebrio molitor* L.) Larvae

**DOI:** 10.3390/molecules29153679

**Published:** 2024-08-03

**Authors:** Radosław Bogusz, Joanna Bryś, Anna Onopiuk, Katarzyna Pobiega, Aneta Tomczak, Przemysław Łukasz Kowalczewski, Katarzyna Rybak, Małgorzata Nowacka

**Affiliations:** 1 Department of Food Engineering and Process Management, Institute of Food Sciences, Warsaw University of Life Sciences—SGGW, Nowoursynowska 159c, 02-776 Warsaw, Poland; katarzyna_rybak@sggw.edu.pl; 2 Department of Chemistry, Institute of Food Sciences, Warsaw University of Life Sciences—SGGW, Nowoursynowska 159c, 02-776 Warsaw, Poland; joanna_brys@sggw.edu.pl; 3 Department of Technique and Food Development, Institute of Human Nutrition Sciences, Warsaw University of Life Sciences—SGGW, Nowoursynowska 159c, 02-776 Warsaw, Poland; anna_onopiuk@sggw.edu.pl; 4 Department of Food Biotechnology and Microbiology, Institute of Food Sciences, Warsaw University of Life Sciences—SGGW, Nowoursynowska 159c, 02-776 Warsaw, Poland; katarzyna_pobiega@sggw.edu.pl; 5 Department of Food Analysis and Biochemistry, Faculty of Food Science and Nutrition, Poznań University of Life Sciences, Wojska Polskiego 28, 60-623 Poznan, Poland; aneta.tomczak@up.poznan.pl; 6 Department of Food Technology of Plant Origin, Faculty of Food Science and Nutrition, Poznań University of Life Sciences, Wojska Polskiego 31, 60-624 Poznan, Poland; przemyslaw.kowalczewski@up.poznan.pl

**Keywords:** edible insects, drying, insect protein, oil properties, bioactive properties, mineral composition, allergen content, microbiological quality

## Abstract

The growing world population necessitates the implementation of appropriate processing technologies for edible insects. The objective of this study was to examine the impact of distinct drying techniques, including convective drying at 70 °C (70CD) and 90 °C (90CD) and freeze-drying (FD), on the drying kinetics, physical characteristics (water activity, color), chemical characteristics (chemical composition, amino acid profile, oil properties, total polyphenol content and antioxidant activity, mineral composition, FTIR), and presence of hazards (allergens, microorganisms) of blanched yellow mealworm larvae. The freeze-drying process results in greater lightness and reduced moisture content and water activity. The study demonstrated that the freeze-dried insects exhibited lower contents of protein and essential amino acids as compared to the convective-dried insects. The lowest content of total polyphenols was found in the freeze-dried yellow mealworm larvae; however, the highest antioxidant activity was determined for those insects. Although the oil isolated from the freeze-dried insects exhibited the lowest acid and peroxide values, it proved to have the lowest PUFA content and oxidative stability. All the samples met the microbiological criteria for dried insects. The results of the study demonstrate that a high temperature during the CD method does not result in the anticipated undesirable changes. It appears that freeze-drying is not the optimal method for preserving the nutritional value of insects, particularly with regard to the quality of protein and oil.

## 1. Introduction

Alternative and sustainable food sources constitute one of the contemporary objectives of research and discussion subject matter in terms of ensuring global food security and counteracting the environmental changes that are taking place. The use of edible insects as a source of many valuable nutrients is one solution that is often pointed out [1,2]. The interest in edible insects has increased since 1 January 2018, when the European Union changed its legislation and recognized insects and their parts as a novel food according to Regulation (EU) 2015/2283 on novel foods [2]. Since then, there has been more interest in insects within the framework of scientific research. One of the most studied species has been the yellow mealworm, which was the first to be included in the list of novel foods of the European Union (in 2021). The yellow mealworm (*Tenebrio molitor* L.) is used both as animal feed and as a source of protein and valuable nutritional ingredients for humans. It is easy and cheap to breed and has a short life cycle and a high reproduction rate [3]. The yellow mealworm contains valuable compounds, including complete protein (47–64% d.m.) with a high digestibility (76–98%) and important amino acids that are often limited in the human diet, such as lysine and methionine. It also contains fat (25–39% d.m.), with up to 77% of this fat being unsaturated fatty acids (oleic, linoleic, α-linolenic acids), carbohydrates (2–14% d.m.), and various minerals and vitamins [2,4,5]. The chemical composition and nutritional value of larvae may vary depending on their diet, life stage, rearing conditions, and processing method. It has been shown that freeze-dried larvae have a lower content of lysine and tryptophan as compared to those dried in the oven and in the sun, but their amino acid composition is balanced [6]. It has also been shown that yellow mealworms may be a source of lecithin with a better chemical composition as compared to soybeans [7]. In addition to their nutritional properties, other beneficial effects on human health resulting from the consumption of yellow mealworms have also been demonstrated, i.e., prebiotic and antioxidant properties, and their extracts also have anti-cancer properties [8].

Insects require proper processing, i.e., blanching, freezing, or drying before consumption. That step is mainly related to safety, as they are not free of microbiological contamination [9,10]. A well-known and widely used pretreatment method is blanching, in which insects are heated either by immersion in hot water or by means of a stream of water steam. Such treatment allows for the inactivation of microorganisms or enzymes responsible for undesirable changes, including darkening. Thermal treatment causes a number of adverse changes, both physical (color and texture) and chemical (protein, fat, and bioactive compounds). However, the destroyed cellular structures improve the course of many processes, such as freezing, drying, and the extraction of various substances [10,11,12].

A common method of food preservation is drying, during which water is removed, which prevents the growth of microorganisms and reduces the course of chemical reactions (enzymatic and non-enzymatic) [10,13]. The most commonly used drying technique is convective drying. Nonetheless, it is one of the worst methods, as it causes undesirable physical (shrinkage, often high hardness and low porosity, and darker color) and chemical (reduction in the quantity and quality of components such as proteins and bioactive components) changes due to prolonged exposure to oxygen and high temperatures [14,15,16]. For this reason, other drying methods are being considered. Freeze-drying (also termed sublimation drying or lyophilization) involves the removal of water from the previously frozen material by means of ice sublimation, omitting the liquid phase [16]. The dried product is of high quality due to its physical (porous structure, good rehydration ability, attractive color), chemical (preservation of valuable nutrients and biologically active compounds), and sensory (appealing taste and aroma) properties. In addition, due to the low water content (up to 2–3%), freeze-dried materials are characterized by limited chemical reactions (enzymatic and non-enzymatic) and high storage stability under properly selected conditions [16,17,18]. However, freeze-dried products are characterized by high hygroscopicity and, therefore, the need to choose an appropriate packaging [18]. Furthermore, the freeze-drying process itself takes a relatively long time and requires complex and expensive devices, which account for the high cost of such a process [17]. 

According to some articles, diverse drying methods impact the quality of yellow mealworms. For instance, Kröncke et al. [19,20] have reported a lower protein content in freeze-dried insects as compared to convective-dried ones. However, the protein solubility of freeze-dried insects is higher. According to Selaledi and Mabelebele [21], freeze-dried yellow mealworms are characterized by a lower content of essential amino acids such as lysine, phenylalanine, threonine, and valine as compared to the convective-dried ones. In addition, a lower content of α-linolenic and oleic acids and a higher content of linoleic acid have been found in the oil isolated from freeze-dried yellow mealworms as compared to the convective-dried ones. It has also been found that the oil from freeze-dried insects exhibits greater oxidative changes as measured by 4-HNE content [20] and acid value [22]. Additionally, lower contents of calcium and magnesium but higher contents of iron and potassium have been found in freeze-dried insects as compared to the convective-dried ones [21]. In turn, Vlahova-Vangelova et al. [22] have shown a higher total viable count and a lower coliform count for freeze-dried yellow mealworms than for convective-dried ones. 

The quality of the dried material is affected by the drying method as well as by the drying parameters applied. Therefore, the purpose of this work has been to assess the impact of the drying method on the quality of the obtained dried insects. For this purpose, the basic chemical composition, amino acid profile, oil properties (fatty acid composition, TAG structure, oxidative stability, acid value, peroxide value), mineral composition, FTIR, allergen content, and microbiological quality have been determined. Furthermore, the drying kinetics, water activity, and color have been analyzed.

## 2. Results and Discussion

### 2.1. The Effect of Blanching and Diverse Drying Methods on Drying Process, Water Activity, and Color of Yellow Mealworm Larvae

This study revealed that the convective method significantly reduced the drying time of yellow mealworm larvae as compared to freeze-drying (Table 1). The greater temperature difference between the surrounding air and the product in convective drying causes the product to heat up faster and the water molecules to evaporate faster [23]. During convective drying at a higher temperature (90 °C), the process was completed 31.4% more quickly. Applying a higher temperature increases the slope of the moisture content chart, thus reducing the drying time (Figure S1). Previous studies have not confirmed the finding that the drying time of bee larvae using the convective method was shorter than that when utilizing freeze-drying [23]. Nonetheless, an increase in drying temperature has been shown to reduce the duration of the process, as evidenced by the freeze-drying of bee larvae [23] and the convective drying of yellow mealworm larvae [24].

A food product’s water activity may be used to determine how much water is present and available, which may affect microbial growth and chemical and enzymatic activities [25]. The freeze-dried insects exhibited lower water activity (0.068) than the convective-dried insects, ranging from 0.220 to 0.225 (Table 1). The results obtained for freeze-dried insects were lower, whilst for convective-dried insects, they were higher than those previously reported in the literature [4,22,26]. Values of water activity lower than 0.6 indicate that dried insects are not susceptible to the growth of microorganisms [25]. Nevertheless, values above 0.2 may facilitate non-enzymatic browning [4].

How the material is processed affects its color, which in turn is an important quality differentiator for consumers [27]. The drying methods significantly influenced the values of the color parameters. Freeze-dried yellow mealworms were characterized by a lighter color and a higher proportion of redness and yellowness (Table 1) compared to convective-dried insects, which was also observed by Lenaerts et al. [4]. Our study also exhibited a lighter color and higher proportions of redness and yellowness of freeze-dried insects as compared to results provided by Selaledi and Mabelebele [21]. From the consumer’s point of view, the color of freeze-dried insects is advantageous, as they are generally more likely to choose products characterized by more red and yellow colors [28]. In turn, the lower lightness values of convective-dried insects are linked to high temperatures, promoting non-enzymatic and thermal browning reactions [4,27]. 

The high value of the a* color parameter and the high browning index (BI) of the freeze-dried yellow mealworm suggest the presence of a large amount of dark pigment [27]. These compounds may be attributed to enzymatic browning due to the presence and activity of enzymes that were not degraded due to the low drying temperature. Endogenous enzymes present in insects may hydrolyze triacylglycerol (TAG) into free fatty acids undergoing oxidation. Fat oxidation products can interact with smaller protein molecules, such as free amino acids, and lead to the formation of brown macromolecules [29].

### 2.2. The Effects of Blanching and Diverse Drying Methods on Chemical Composition of Yellow Mealworm Larvae

The chemical composition of dried yellow mealworms is presented in Table 2. The compound found in the highest amounts was protein, and its content was dependent on the drying method. A significantly lower protein content (37.57 g/100 g d.m.) was determined in freeze-dried insects. This may have been due to protein denaturation during freezing as a result of mechanical damage resulting from the formation of ice crystals, as observed by Qui et al. [30] for freeze-dried shiitake mushrooms. It appears that during the freezing process, proteins and chitin may undergo structural changes that may affect the protein content in the sample. The formation of ice crystals may damage the chitin structure, adversely affect the chitin nitrogen content, and lead to underestimation of the protein content. Furthermore, throughout freezing, the contents of some nitrogen-containing compounds (e.g., choline and ortho-phosphocholine) may increase due to their release from the structure of phospholipids [31]. Being water-soluble substances, they may have been removed to a greater extent during freeze-drying (greater water loss as compared to convective drying), resulting in a lower quantified protein content. In addition, protein proteolysis may have occurred as a result of endogenous proteolytic enzymes found naturally in insects [31]. Another explanation could be related to the cold denaturation of the protein, which unfolds the polypeptide chain and exposes the internal non-polar groups of the protein to water [32]. However, our theory requires additional advanced research to confirm it.

The drying method did not cause any changes in the contents of fat and ash, which was also observed in the study conducted by Kröncke et al. [20]. Our results are in line with those provided by Kröncke et al. [19,20], who also demonstrated a lower protein content for yellow mealworms dried by means of freeze-drying as compared to other drying methods (microwave drying, vacuum drying, rack oven drying). Nonetheless, many studies have shown the similar nutritional composition of dried insects, regardless of the drying method chosen [4,9,21]. 

### 2.3. The Effects of Blanching and Diverse Drying Methods on Amino Acid Profile of Yellow Mealworm Larvae

The amino acid composition determines the quality of protein, which is important in terms of human nutrition [33]. The drying method used affected the amino acid profile of blanched and dried yellow mealworms (Table 3). All materials were characterized by a lower content of essential amino acids compared to the content of non-essential amino acids. Statistically significant differences were noted in the content of essential amino acids phenylalanine and valine and non-essential amino acids serine, glycine, alanine, tyrosine, and arginine. In most cases, the lowest content of those amino acids was found for the Tm_70CD sample. Among the essential amino acids important in human nutrition, the major ones were leucine and lysine, followed by valine and threonine. Insects that were convective-dried at 90 °C (Tm_90CD) were characterized by the highest content of essential amino acids among the samples tested and were the most valuable in terms of incorporation into the human diet (Table 3). 

Our results are in line with those provided by Selaledi and Mabelebele [21], who found a lower content of most amino acids in freeze-dried insects as compared to convective-dried ones. Nevertheless, our study has exhibited a higher content of lysine, isoleucine, leucine, and phenylalanine as compared to the published studies [21,34]. Furthermore, due to the presence of lysine, insects may serve as an ingredient in lysine-poor food such as cereals, bakery products, and pasta [35,36].

### 2.4. The Effects of Blanching and Diverse Drying Methods on Oil Properties of Yellow Mealworm Larvae

#### 2.4.1. Fatty Acid Composition and Positional Distribution of Triacylglycerols

Fatty acid composition is one of the basic quality characteristics used for determining the techno-functional properties and nutritional value of fat and oil. Yellow mealworm oils contained up to 76.86% unsaturated fatty acids, with a larger percentage of monounsaturated fatty acids (MUFAs), at a level of 52.74–56.05%, than polyunsaturated fatty acids (PUFAs), at a level of 18.15–21.97% (Table 4). The unsaturated fatty acids were mainly represented by oleic acid and linoleic acid. The tested oils contained a small percentage of α-linolenic acid, not exceeding 1%. It was also observed that the oil isolated from freeze-dried insects was characterized by a significantly higher percentage of oleic acid and by significantly lower percentages of linoleic acid and α-linolenic acid as compared to the oil extracted from convective-dried insects. Among the oils under analysis, the freeze-dried insect oil presented with the highest percentage (24.21%) of saturated fatty acids (SFAs), especially palmitic acid (C16:0) and stearic acid (C18:0). Those results may be associated with these samples presenting with the lowest content of water (Table 2) and water activity (Table 1), which facilitate the oxidation process of unsaturated fatty acids. In the near absence of water, the hydration spheres that form around metal ions are not sufficient to reduce their catalytic ability to trigger free radical formation and increase the oxidation rate [37]. The fatty acid composition of the tested yellow mealworm oils was comparable to the results provided in previous studies [38,39,40]. A higher percentage of α-linolenic acid (1.52–1.67%) [21] and linoleic acid at levels of 23.15–30.37% [4] and 25.73% [41] were demonstrated in the related literature. 

In order to evaluate the prospective nutritional value of yellow mealworm oils, theoretical health indices related to the possible health-promoting mitigation of cardiovascular disease risk, such as an atherogenicity index (IA), a thrombogenicity index (TI), and a hypocholesterolemic/hypercholesterolemic ratio (HH), have been calculated [42]. In general, a lower AI (<1.0) and TI (<0.5) and a higher HH (>1.5) of the tested oil indicate a health-promoting effect, accounting for a reduction in the accumulation of atherosclerotic plaque and the level of total cholesterol (together with LDL fraction) and phospholipids [42,43]. Among the analyzed oils, the sample isolated from Tm_70CD has suggested the best possible nutritional value due to its presentation of the lowest TI value and the highest HH value (Table 4). The indices calculated for the isolated oils are comparable to those of chicken fat [44], lamb fat [45], and herring and carp oil [46]. The n-6/n-3 fatty acids ratio also gives some information about the health-promoting effect of oil, including the pro-inflammatory effect and lipids’ metabolic pathway. The yellow mealworm oils tested within the framework of the study are characterized by a higher ratio of n-6/n-3 acids than the recommended threshold (between 2:1 and 5:1), which may promote an incidence of diseases such as inflammatory bowel disease, diabetes, obesity, or asthma. The comparable or even higher ratio of n-6/n-3 fatty acids of yellow mealworm oil has been demonstrated in previous studies [21,38,47]. 

The distribution of selected fatty acids in the internal (*sn*-2) position of TAG molecules of the tested yellow mealworm oils is presented in Table 5. Unsaturated fatty acids (oleic acid and linoleic acid) occupied the internal (*sn*-2) position of TAG molecules, while saturated fatty acids (myristic acid, palmitic acid, stearic acid) were located mainly in the external (*sn*-1,3) positions of TAG molecules, also shown in Table 5. Among the saturated fatty acids, stearic acid accounted for the largest percentage share in the *sn*-2 position of TAG molecules. The oil isolated from convective-dried insects at 70 °C accounted for a significantly lower percentage share of both oleic acid and linoleic acid in the *sn*-2 position of TAG molecules. By and large, the fatty acid positional distribution of yellow mealworm oils is not favorable in terms of their bioavailability, because the long-chain saturated fatty acids released from the external (*sn*-1,3) positions of TAG molecules through hydrolysis form insoluble calcium salts and are removed during defecation from the body [48].

#### 2.4.2. Oxidative Stability, Acid Value, and Peroxide Value

Oxidative stability is one of the most important properties of fat, linked to its susceptibility to oxidation processes [48]. The results regarding the oxidative stability of oils extracted from dried insects are presented in Table 6. Oils extracted from convective-dried insects are characterized by a statistically longer oxidative stability than the oil extracted from insects dried by means of a freeze-drying method. That result may be related to the higher content of antioxidant compounds (see Table 7) and the reduced activity of endogenous enzymes that were inactivated due to the high drying temperatures. Their activity was not inhibited by the antioxidant compounds present in the lipid fraction and could, therefore, have limited the oil oxidation during the measurement.

The quality of oil and its susceptibility to oxidation processes may also be evaluated by means of fatty values such as the acid and peroxide values. The highest acid value was found for the oil extracted from convective-dried insects at 70 °C (Table 6), and this acid value (16.72 mg KOH/g) exceeds the recommended threshold for oils, as referred to in the Codex Alimentarius (4 mg KOH/g of oil), which suggests that the oil has undergone oxidation and degradation, and cannot be consumed. There is no statistical difference between the acid values of the oils isolated from the other samples. The results of the acid value of those tested oils are close to the recommended value for oils, as referred to in the Codex Alimentarius. To date, very discrepant acid values for insect oils are available. For example, Son et al. [38] have provided a lower acid value (2.6 mg KOH/g of oil) for the oil isolated from convective-dried yellow mealworms, while Lee et al. [39] have reported a higher acid value (104.8 mg KOH/g of oil) for the oil from freeze-dried yellow mealworms.

The lowest peroxide value was determined for the oil extracted from freeze-dried insects. The peroxide values for the oil isolated from convective-dried insects are significantly higher, demonstrating an increasing trend with increasing drying temperature. The peroxide values for all the tested insect oils do not exceed the level referred to in the Codex Alimentarius (15 meq O_2_/kg of oil). However, the peroxide value for the oil from convective-dried insects exceeds the level stipulated in the Commission Implementing Regulation (EU) 2021/882 (5 meq O_2_/kg of oil). As far as the comparison of the oil from freeze-dried mealworms to oils isolated from convective-dried insects is concerned, lower peroxide values, at a level of 3.5 meq O_2_/kg of oil for the oil isolated from convective-dried yellow mealworms [38] and at a level of 1.0 meq O_2_/kg of oil for the oil from freeze-dried yellow mealworms [39] have been reported.

The differences in the results of acid and peroxide values in this study may be related to diversified drying and the properties of other compounds and possible interactions. Generally, the low water activity of the freeze-dried sample (Table 1) should facilitate the oxidation process [37]. Interestingly, it is the higher water activity of the sample (at a level of 0.2, which is within the range ensuring the greatest stability) that caused greater oxidative changes. The exposure to oxygen and high temperatures during convective drying promoted the oxidation process and the formation of primary oxidation products, as indicated by a higher peroxide value [22,49]. The higher acid value of the oil isolated from insects dried at 70 °C may be related to the greater lipase activity [50]. The sample did not reach the drying air temperature, so the temperature did not degrade endogenous lipases, which in turn facilitated the hydrolysis of TAG molecules into free fatty acids and, thus, impacted the acid value.

### 2.5. The Effects of Blanching and Diverse Drying Methods on the Bioactive Properties of Yellow Mealworm Larvae

Bioactive compounds, such as polyphenols, are of significant importance to human health. Those compounds reduce oxidative stress and protect cells from damage or death caused by reactive oxygen species [13]. A significantly higher total polyphenol content was found in the convective-dried than in the freeze-dried insects (Table 7). This may have been related to the inhibition of enzymes and other chemical reactions, resulting in less need for antioxidant compounds such as polyphenols [51], as well as the loss of those compounds along with the effects of sublimating ice crystals during the freeze-drying process [52]. The drying temperature during the convective process had no significant effect on the total polyphenol content; nevertheless, the insects dried at a higher temperature exhibited a higher total polyphenol content. A study conducted by Baek et al. [53] has proven that there is a higher total polyphenol content for freeze-dried yellow mealworms (9.2 mg GAE/g) as compared to the hot air-dried ones (5.2 mg GAE/g). The obtained results are, in fact, up to 13 times higher in comparison to the findings of our study. The differences in results are related to the fact that insects contain greater amounts of gallic acid as compared to chlorogenic acid [54]. 

Despite the lower total polyphenol content of the freeze-dried insects, a significantly higher antioxidant activity (both DPPH and ABTS assays) was observed in them as compared to convective-dried insects (Table 7). Since bioactive components are sensitive to high temperatures [13,51], the lower temperature during freeze-drying preserves their better quality and, hence, the higher antioxidant capacity of the Tm_FD sample. Lucas-González et al. [55] also found a higher DPPH antioxidant activity for freeze-dried house crickets (7.1 mg TE/g) as compared to the convective-dried ones (2.8 mg TE/g). In turn, the effect of the drying method was not proven by Vlahova-Vangelova et al. [22], who observed a comparable DPPH antioxidant activity for freeze- and convective-dried yellow mealworms. In addition, other compounds may also have influenced the antioxidant activity. The antioxidant properties could be attributed to oleic acid [56], the content of which, in the case of freeze-dried insects, was found to be the highest. The presence of peptides, especially those of low molecular weight, may have a similar effect to antioxidant compounds, too [25,57]. 

**Table 7:** Total polyphenol content and antioxidant activity (ABTS and DPPH assay) of blanched yellow mealworm larvae dried by means of the convective method at 70 °C (Tm_70CD) and 90 °C (Tm_90CD) and the freeze-drying (Tm_FD) method.

	Tm_70CD	Tm_90CD	Tm_FD
Total polyphenol content (mg chlorogenic acid/100 g d.m.)	77.83 ± 2.03 b ^1^	76.06 ± 0.83 b	73.00 ± 1.14 a
ABTS (mg TE/g d.m.)	4.34 ± 0.15 b	3.34 ± 0.03 a	8.10 ± 0.44 c
DPPH (mg TE/g d.m.)	5.38 ± 0.26 b	4.21 ± 0.10 a	5.98 ± 0.01 c

^1^ Different letters within the same row indicate a significant difference between samples (Tukey’s HSD, *p* < 0.05).

### 2.6. The Effects of Blanching and Diverse Drying Methods on the Mineral Composition of Yellow Mealworm Larvae

Insects are increasingly gaining interest also due to their mineral composition, and they may be a good source of macrominerals (Ca, Mg, K) and microminerals (Fe, Se, Zn). The mineral composition of dried yellow mealworm is summarized in Table 8. The content of most minerals was found to be higher in insects dried by means of the convective method. The most significant differences between the convective-dried and freeze-dried samples were determined for K, Mn, Cu, and Zn. Significantly higher levels of those minerals were found in insects dried with the CD method as compared to those dried with the FD method. The contents of Fe and Se were found to be comparable in all dried yellow mealworms. Differences in the mineral contents may be due to variations in the water content, which affects the concentration of the tested minerals, as well as the partial removal of minerals with water during drying. The content of divalent minerals is related to the protein content [58], so the varying amounts of protein could have affected the changes in the content of those minerals. 

Mineral deficiencies in a human diet are the most frequently related to Ca, Mg, and Fe [59]. The reference intake of Ca for an adult is 950 mg. Consuming 100 g of dried yellow mealworms, regardless of the method chosen, exceeds the daily requirements by about 6.8–7.7%. Moreover, 100 g of dried insects provides about 87.0–101.7% of the daily adequate intake for Mg and about 45.2–57.8% of the daily population reference intake for Fe, regardless of the method chosen. Mihaly Cozmuta et al. [47] found higher contents of Na (255.4 mg), Ca (225.1 mg), Mg (324.4 mg), Zn (53.1 mg), and Fe (27.2 mg) and a lower content of K (872.1 mg) in yellow mealworm powder as compared to our study. The available information suggests that insects may have higher Ca, Zn, Cu, and Mn contents than traditional slaughtered animal meat. For example, the study conducted by Pistón et al. [60] has shown that beef cuts have a lower content of Zn (9.5–24.6 mg) and Mn (0.03–0.06 mg), and a similar or higher content of Fe (5.6–11.3 mg), as compared to the results for dried insects in this study. Oz et al. [58] found higher contents of Mg (687.1–836.1 mg), Ca (926.8–1083.3 mg), Fe (51.0–58.0 mg), Zn (316.8–381.6 mg), and K (9958.4–12306.0 mg) but a lower content of Mn (0.57–0.70 mg) for beef steaks cooked by means of diverse methods. Therefore, in our opinion, it is plausible to conclude that insects may serve as an ingredient in various food products to increase their mineral content.

### 2.7. The Effects of Blanching and Diverse Drying Methods on FTIR Spectra of Yellow Mealworm Larvae

**Figure 1:**
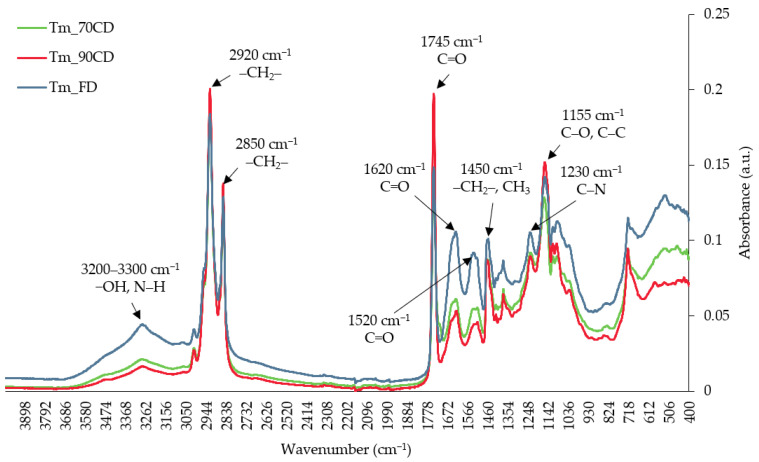
FTIR spectra of blanched yellow mealworm larvae dried by means of the convective method at 70 °C (Tm_70CD) and 90 °C (Tm_90CD) and the freeze-drying (Tm_FD) method.

Fourier transform infrared spectroscopy (FTIR) is a popular method used to determine molecular structure and to identify and quantify functional groups. Absorption of infrared radiation is related to the frequencies of vibration of bonds between the atoms in a molecule [61,62]. The obtained FTIR spectra exhibited similar patterns but with different absorbances for different processing parameters (Figure 1). A weak stretching vibration visible in the region of 3200 to 3300 cm^−1^ is related to the hydroxyl group (−OH) of water molecules, amino acids, phenolic compounds, or carbohydrates [63,64] and the N–H bond of the amide A group [61]. A weak peak at a wavenumber of 3000 cm^−1^ may be responsible for the stretching vibration of the acetamide functional group of chitin [65,66]. Strong peaks at wavenumbers of 2920 cm^−1^ and 2850 cm^−1^ are related to the asymmetric and symmetric, respectively, stretching vibrations of the methylene group (–CH_2_–) [67], and correspond to the aliphatic chains of lipids [62] and chitin [66]. Moreover, a strong peak at a wavenumber of 2920 cm^−1^ also indicates a stretching vibration of the C–H bond originating from the amide B group [61]. A strong peak at a wavenumber of 1745 cm^−1^ is related to the stretching vibration of the carbonyl group (C=O) of lipids [62,66]. A medium peak observed at 1620 cm^−1^ is due to the stretching vibration of the carbonyl group (C=O) of the amide group from amide I [61]. A medium peak visible at a wavenumber of 1630 cm^−1^ and 1520 cm^−1^ may be linked to bioactive ingredients such as phenolic compounds [13]. A weak peak around 1520 cm^−1^ identifies amide II, while a medium peak at 1230 cm^−1^ corresponds to amide III [61,62]. A weak peak at a wavenumber of 1450 cm^−1^ is linked to the bending vibrations of the methylene group (–CH_2_–) and the methyl group (CH_3_) and corresponds to lipids and proteins [62] as well as polysaccharides [66]. The spectral region in the range of 1200 to 900 cm^−1^ (fingerprint region) is generally related to the stretching vibrations of the C–C, C–O–C, and C–O bonds, and the bending of the C–O–H bond of different carbohydrate groups [61,62]. The region below the wavenumber of 900 cm^−1^ corresponds to conformational changes in the tested material, in which each organic compound exhibited unique molecular vibrations [68].

The highest differences between the samples were observed in the ranges of 3600–3000 cm^−1^ and 1700–1200 cm^−1^ (Figure 1). A higher absorption in these ranges was observed for yellow mealworms dried with the freeze-drying method, which may be related to the decreased damage to the structure and bonds in the molecules than when high temperatures are applied during convective drying. The protein’s secondary structure and, indirectly, its quality correspond mainly to amide I and amide II due to the presence of a sensitive C=O bond [69]. The freeze-dried insects were characterized by a greater absorbance intensity for these peaks than convective-dried insects, which may indicate their better protein secondary structure. However, given the lower protein content (Table 2), it is more probable that the carbonyl groups, which are products of protein and lipid oxidation, were responsible for this phenomenon [70]. 

### 2.8. The Effects of Blanching and Diverse Drying Methods on the Allergen Content of Yellow Mealworm Larvae

Consuming insects may pose a risk for those with crustacean and mollusk allergies due to the potential for cross-reactions with allergens found in other arthropods, such as tropomyosin and arginine kinase. Previous studies have reported allergic reactions in individuals with shellfish allergies after they have consumed insect-based products [71]. According to the EFSA, the ingestion of yellow mealworms may result in primary sensitization to their proteins. Additionally, allergens from the substrate on which the insects were raised may also be present in the insects and trigger an allergic reaction [72]. Given the limited data on this topic, paying special attention to this issue is crucial because of the potential risk it poses to consumers. 

Tropomyosin is a particularly noteworthy allergenic protein in terms of its resilience to temperature fluctuations. The highest content of crustacean tropomyosin was observed in freeze-dried insects, with a content comparable to those reported in the literature for yellow mealworm larvae [57]. Drying with the convective method significantly reduced crustacean allergens (Table 9). Insects dried at a higher temperature (90 °C) showed a significantly higher content than those dried at 70 °C. This may be linked to the effect of heat treatment on protein structure and solubility, as demonstrated by Broekman et al. [71]. The same study found that heat treatment did not reduce the content of detectable allergens in the heat-treated yellow mealworms but changed their solubility, including that of denatured allergens. Therefore, differences may indicate increased allergenicity of mealworm samples following more intensive heat treatment [71,73].

Even after heating and cooling during food processing, tropomyosin proteins may retain their conformational structure. Chemical processes, such as the Maillard modification, may increase the allergenicity of tropomyosin. Studies of allergenic arginine kinases from crustaceans suggest that these enzymes are unstable at temperatures between 40 and 80 °C. In this temperature range, partial unfolding may occur, revealing new epitopes that can enhance IgE reactivity. Once the temperature exceeds 80 °C, arginine kinase undergoes complete unfolding, decreasing its immunogenicity [74]. Furthermore, heat treatment may affect the initial change in allergen protein structures and their denaturation. For crustacean allergens, it is evident that higher temperatures lead to further degradation. However, for mollusk allergens, additional heat treatment resulted in the detection of more allergens [75].

### 2.9. The Effects of Blanching and Diverse Drying Methods on the Microbiological Quality of Yellow Mealworm Larvae

Due to the various methods of feeding and transporting as well as processing edible insects, it is necessary to monitor their basic microbiological parameters, such as the total count of microorganisms and the total count of fungi. Edible insects may be contaminated with foodborne pathogens both on their surface and inside their digestive tracts [76,77]. Therefore, the selection of processing parameters is crucial for edible insect products to be approved for consumption.

The raw yellow mealworm larvae were characterized by a total count of microorganisms of 5.63 log CFU/g, and the number of *Enterobacteriaceae* was at the level of 3.27 log CFU/g, including *E. coli*—2.88 log CFU/g and 3.86 log CFU/g of aerobic spore-forming bacteria. The obtained dried insects had a proven good microbiological quality. The tested edible insects were characterized by a low count of microorganisms, which may have been due to the blanching used for pretreatment purposes (Table 10). The total number of fungi was found to be in the range of 1.5–2.3 log CFU/g. It was observed that the use of convective drying at a higher temperature (90 °C) may have contributed to a reduction in the number of fungi, which was probably caused by a reduction in the number of yeasts; there was a constant number of molds (the spores of which may withstand the increased drying temperature). The presence of aerobic spore-forming bacteria with counts of 1.6–1.7 log CFU/g was also observed in the samples, which may indicate contamination with *Bacillus* spp. No differences were found in terms of the aerobic spore-forming bacteria count in the samples dried by means of diverse methods. The tested dried insects were characterized by a water activity below 0.2 (Table 1), which indicates the lack of possibility of microbial growth. In such conditions, bacterial and mold spores could survive, hence the presence of aerobic spore-forming bacteria in the samples. In the tested dried yellow mealworm larvae, the presence of pathogenic food-borne bacteria, such as *L. monocytogenes, Salmonella* spp., was not observed, which proves their good microbiological quality and their suitability for consumption. Furthermore, all the tested insects’ exhibited microbial load remained within the limits set forth in the Commission Implementing Regulations [78,79].

The study by Vandeweyer et al. [80] has shown that 40 s of blanching and microwave drying significantly reduces the total number of bacteria, *Enterobacteriaceae*, lactic acid bacteria, and yeasts and molds, while aerobic endospores may be slightly reduced [80]. The research conducted by Ribeiro et al. [9] has shown that blanching and drying reduces the total viable count, *Enterobacteriaceae*, and aerobic endospores, except for yeasts and molds of yellow mealworms. In turn, the research carried out by Vlahova-Vangelova et al. [22] has not indicated any differences between the content of the total number of microorganisms in yellow mealworms depending on the drying method. The same studies have shown that dried larvae are not microbiologically contaminated by *E. coli*, molds, and yeasts. Previous research has shown that the application of just a drying treatment is not as effective in reducing the microorganism load [57,77]. Furthermore, the blanching process at 60 °C for 5 min appears to be the minimal time/temperature combination required to achieve a substantial reduction in microbial load [12,40].

## 3. Materials and Methods

### 3.1. Material

The alive yellow mealworm (*Tenebrio molitor* L.) larvae were delivered by a local Polish producer (Cirwins, Kamień Duży, Poland) and then stored under refrigerated conditions at a temperature of 4 ± 1 °C for 24 h of fasting until the experiments were performed. Before experiments, the material was gently washed by dipping it in tap water and then dried with filter paper. The raw material consisted of 63.4% moisture, 16.9 g/100 g w.m. (wet mass) of protein, 1.6 g/100 g w.m. of fat, and 1.3 g/100 g w.m. of ash. 

### 3.2. Technological Treatment

#### 3.2.1. Blanching

Blanching (BL) treatment was conducted directly in boiling tap water at a temperature equal to 98 °C for 5 min with a water-to-larvae ratio equal to 10:1. After treatment, the larvae were strained on a sieve, cooled in a stream of cold water for 30 s, and then dried with filter paper. The blanching was done in duplicate.

#### 3.2.2. Drying

For convective drying, the material (about 100 g) was dried using a prototype laboratory dryer (Warsaw, Poland) with the following parameters: temperature of 70 °C or 90 °C, air velocity of 2 m/s parallel to the material layer, and sieve load of 0.83 kg/m^2^. 

For freeze-drying, the material (about 100 g) was first frozen using a Shock Freezer HCM 51.20 (Irinox, Treviso, Italy) at a temperature of −40 °C for 5 h, and then freeze-dried using a Gamma 1–16 LSC laboratory lyophilizer (Martin Christ Gefriertrocknungsanlagen GmbH, Osterode am Harz, Germany) with the following parameters: a shelf temperature of 40 °C, a condenser temperature of −55 °C, and a pressure of 63 Pa.

For convective drying, changes in the mass of the sample were recorded continuously every 5 min, whilst for freeze-drying, this was done continuously every 5 min for the first 120 min and every 15 min for the remaining time (using an SWL0125 system (Mensor, Warsaw, Poland) placed in the drying chamber and connected to a specially designed scale, which was placed outside the freeze-dryer). Drying processes were continued until the material achieved a constant weight and were conducted in duplicate for each sample. After the process, the dried material was carefully packaged in air and light barrier PET12/AL8/PE100 bags (Pakmar, Warsaw, Poland). Directly before analysis, the dried insects were ground using an analytical grinder, IKA A11 basic (IKA-Werke GmbH, Staufen, Germany).

Drying curves were plotted as dimensionless water content (MR) as a time function. The relative moisture ratio was calculated based on the following equation:

(1) $$\mathrm{MR}=\frac{u_{\tau }}{u_{0}},$$

where *u_τ_* is the moisture content during a given moment of the drying process (g water/g d.m.); *u*_0_ is the initial moisture content (g water/g d.m.). 

### 3.3. Water Activity

The water activity of dried insects was measured in triplicate for each sample using a HygroLab C1 hygrometer (Rotronic, Bassersdorf, Switzerland) at 24 ± 1 °C [76].

### 3.4. Color Measurement

The color was measured in reflected light using a CM-5 chromometer (Konica Minolta, Osaka, Japan) in the CIE L*a*b* system, CIE Standard Illuminate D65, CIE: 2° Standard Observer, di: 8° (diffuse illumination/8° viewing angle), and 30 mm measurement area in 15 repetitions. The browning index (BI) was calculated as follows:

(2) $$\mathrm{BI}=\frac{100\cdot \left[\left(\frac{a^{*}+1.75L^{*}}{5.645L^{*}+a^{*}-0.3012b^{*}}\right)-0.31\right]}{0.17},$$

### 3.5. Chemical Composition

The chemical composition of fresh and dried insects was performed following the previously described methods [4,76]. In brief, the fat content was determined by the Soxhlet method using a rapid Soxhlet extraction apparatus Soxtherm SE 416 (Gerhardt, Königswinter, Germany). The protein content was determined by the Kjeldahl method using a fully automated VAPODEST 50s distillation system (Gerhardt, Königswinter, Germany), and a nitrogen-to-protein conversion factor of 4.76 was used. The ash was determined using a muffle furnace, the Protherm PLF120/5 (Protherm Furnace, Ankara, Turkey), at 550 °C until a constant weight was achieved. The moisture content was determined by the oven method using a DHG-9240A oven (Yiheng Inc., Shanghai, China) for 17 h at a temperature of 105 °C until a constant weight was achieved [4,76].

### 3.6. Amino Acid Profile

The analysis of the amino acid profile was performed with the Ultra-High-Performance Liquid Chromatography (UHPLC) method. The total amino acid content was analyzed after acid hydrolysis (110 °C, 23 h) and oxidative hydrolysis (4 °C, 16 h and 100 °C, 2 h) following the AOAC method [81]. Acid hydrolysis allows the determination of most protein amino acids [L-alanine (Ala), L-arginine (Arg), L-aspartic acid (Asp) + L-asparagine (Asp), L-glutamic acid (Glu) + L-glutamine (Gln), L-leucine (Leu), L-lysine (Lys), L-serine (Ser), L-threonine (Thr), L-tyrosine (Tyr), L-valine (Val), L-histidine (His), L-isoleucine (Ile), L-phenylalanine (Phe), L-proline (Pro), glycine (Gly)]. In turn, oxidative hydrolysis is used to determine sulfur amino acids: L-methionine (Met) and L-cysteine (Cys). After hydrolysis, the samples were subjected to amino acid derivatization (AccQ•Tag reagents, no. 186003836, Waters, Milford, MA, USA) according to the Waters protocol. This process is based on the reaction in an alkaline borate buffer of primary and secondary amino groups and 6-aminoquinolyl-N-hydroxysuccinimidyl carbamate (ACQ), resulting in highly stable urea derivatives of amino acids, fluorescing at 395 nm after excitation at 250 nm. For all amino acids, derivatization proceeds within a minute at room temperature. In the case of tyrosine, the sample was additionally incubated at 55 °C for 10 min. The analysis of amino acids was performed using the UHPLC chromatograph (Shimadzu Nexera 2.0, Kyoto, Japan) equipped with a binary solvent manager, column heater, PDA detector (Kyoto, Japan), and a separation column, AccQ-Tag Ultra C18 (2.1 × 100 mm, particles of 1.7 μm, Waters Corporation, Milford, MA, USA), which was dedicated to amino acid analyses. A mobile phase flow rate of 0.6 mL/min, a non-linear separation gradient, and a column temperature of 55 °C were applied. An amount of 1 µL of the sample was injected for amino acid determination [82]. A calibration curve was prepared using a commercial amino acid standard, AAS18 (Merck, Darmstadt, Germany). The individual amino acid contents were given in mg/g of protein.

### 3.7. Oil Properties

#### 3.7.1. Extraction Procedure

The oil was extracted using the Folch method described by Kozłowska et al. [83]. In brief, ground insects were stirred with a chloroform/methanol solution, heated, and, after cooling intensively, stirred with an additional chloroform portion. To the resulting filtrate, 0.1 mol/L potassium chloride solution was added. After the overnight phase separation, the lower phase was collected, dried with anhydrous magnesium sulfate, and filtered. The chloroform was then evaporated from the filtrate. The oil was extracted in duplicate.

#### 3.7.2. Fatty Acid Composition

The fatty acid methyl esters (FAMEs) were formed from extracted oil according to the ISO 12966-2:2017 method [84]. FAMEs were analyzed using a YL6100 GC gas chromatograph with a flame ionization detector (FID) and equipped with a BPX 70 capillary column (60 m × 0.25 mm inner diameter, 0.25 μm film thickness, SGE Analytical Science, Milton Keynes, UK). The separation of FAMEs was determined with the following parameters of the oven: 70 °C for 5 min, 15 °C/min to 160 °C, 1.1 °C/min to 200 °C, 200 °C for 12 min, 30 °C/min to 225 °C, and 225 °C for 1 min. The injector and detector temperatures were set at 225 °C and 250 °C, respectively [85]. Each sample was measured in triplicate. FAMEs were identified by comparison of their retention times with the reference standard (Supelco 37 Component FAME mix, Sigma-Aldrich GmbH, Schnelldorf, Germany). The percentage contents of given fatty acids were determined based on the area in the chromatogram.

#### 3.7.3. Fatty Acids Distribution in Triacylglycerols

The fatty acid distribution in triacylglycerols (TAGs) was determined by a partial hydrolysis method using a regiospecific pancreatic lipase due to its ability to hydrolyze the ester bonds in the *sn*-1,3 positions of TAG [85]. To 0.1 g of oil samples, 8 mL of 1M TRIS–HCl at pH 8.0, 0.5 mL of CaCl_2_ (2.2% *w*/*w*), and 0.2 mL of bile salts (1% *w*/*w*) were added. After 30 s of stirring, 20 mg of lipase from porcine pancreas (Merck KGaA) was added and remixed for 30 s. Then, the samples were incubated at 40 °C in a water bath for 10 min, and 1 mL of 6M HCl and 4 mL of diethyl ether were then added, and the samples were stirred for 1 min. Subsequently, the obtained mixtures were centrifuged for 5 min at 4000 rpm. The separation of *sn*-2 monoacylglycerols (*sn*-2 MAGs), products of enzymatic hydrolysis dissolved in diethyl ether, was performed by a preparative thin layer chromatography (TLC) technique using silica gel plates and a solvent solution (hexane/diethyl ether/acetic acid in the ratio 50:50:1 *v*/*v*/*v*). The *sn*-2 MAG layer was scraped off, extracted using 1 mL of diethyl ether, and centrifuged [86]. The ether layer was collected and evaporated, dissolved using hexane, and methylated. The *sn*-2 MAG fatty acid composition was determined following the procedure described for fatty acid composition.

#### 3.7.4. Oxidative Stability

The oxidative stability of the extracted oil was assessed by a Pressure Differential Scanning Calorimetry (PDSC) method using a DSC Q20 TA Instrument (Newcastle, WA, USA) coupled with a high-pressure cell. About 3 mg of oil sample was placed in an aluminum pan. The analysis was determined with the following parameters: a temperature of 140 °C and an oxygen pressure of 1400 kPa [85]. Analysis was performed in triplicate for each sample.

#### 3.7.5. Acid Value

The acid value of the extracted oil was determined by a titration method. The oil sample was dissolved in 50 mL of a mixture of ethanol and diethyl ether (1:1, *v*/*v*), and the free fatty acids present in the mixture were then titrated with a 0.1 M ethanolic potassium hydroxide solution using an automatic titrator, TitraLab AT1000 (HACH LANGE, Wrocław, Poland). Analysis was performed in triplicate for each sample.

#### 3.7.6. Peroxide Value

The peroxide value of the extracted oil was determined by a titration method. The oil sample was dissolved in 25 mL of a mixture of chloroform and acetic acid (2:3, *v*/*v*) and mixed with 1 mL of a solution of potassium iodide. The prepared mixture was stirred for 1 min. After magnetic stirring, the sample was stored in a dark place for 5 min. The iodine released was then titrated with a 0.001 M sodium thiosulfate solution using an automatic titrator, TitraLab AT1000 (HACH LANGE, Wrocław, Poland). Analysis was performed in triplicate for each sample.

#### 3.7.7. Health Indices

The atherogenicity index (AI), the thrombogenicity index (TI), and the hypocholesterolemic/hypercholesterolemic (HH) ratio were calculated [87] as follows:

(3) $$AI=\frac{C12:0+4\times C14:0+C16:0}{\sum UFA}$$

(4) $$TI=\frac{C14:0+C16:0+C18:0}{\frac{\sum MUFA}{2}+\frac{\sum n-6PUFA}{2}+(3\times \sum n-3PUFA)+\frac{n-3PUFA}{n-6PUFA}}$$

(5) $$HH=\frac{C18:1n-9+\sum PUFA}{C12:0+C14:0+C16:0}$$

### 3.8. Bioactive Properties

#### 3.8.1. Extract Preparation

The ground insects (2 g) were weighed into the falcon and 10 mL of 80% ethyl alcohol was added. Extraction was performed for 12 h at room temperature on a Multi Reax shaker (Heidolph Instruments, Schwabach, Germany) in the darkness. The solution was centrifuged using a MegaStar 600 laboratory centrifuge (VWR, Leuven, Belgium) for 2 min at 4350 rpm. For each sample, the extraction procedure was done twice.

#### 3.8.2. Total Polyphenol Content

The total polyphenol content was measured according to the procedure described in detail by Bogusz et al. [57]. An amount of 10 µL of the extract and 40 µL of 5-fold-diluted Folin–Ciocalteu reagent were mixed in 96-well plates, and then 250 µL of 7% sodium carbonate solution was added after 3 min. After incubation for 60 min at 25 °C, absorbance at 750 nm was measured using a Multiskan Sky plate reader (Thermo Electron Co., Waltham, MA, USA). Each sample was measured in triplicate. A calibration curve in the range of 0 to 100 µg/mL was prepared for chlorogenic acid (Sigma-Aldrich, Saint Louis, MO, USA). The total polyphenol content was expressed in mg of chlorogenic acid/100 g of sample dry matter.

#### 3.8.3. ABTS Assay Antioxidant Activity

The antioxidant activity of dried insects to scavenge the ABTS^•+^ cation radicals was measured according to the procedure described in detail by Bogusz et al. [57]. An amount of 10 µL of the extract and 250 µL of the radical working solution were mixed in the 96-well plates. After incubation for 6 min at 25 °C, absorbance at 734 nm was measured using a Multiskan Sky plate reader (Thermo Electron Co., Waltham, MA, USA). Each sample was measured in triplicate. Antioxidant activity was expressed in mg of Trolox/g of sample dry matter.

#### 3.8.4. DPPH Assay Antioxidant Activity

The antioxidant activity of dried insects to scavenge the DPPH^•^ radicals was measured according to the procedure described in detail by Bogusz et al. [57]. An amount of 10 µL of the extract and 250 µL of the radical working solution were mixed in the 96-well plates. After incubation for 30 min at 25 °C, absorbance at 515 nm was measured using a Multiskan Sky plate reader (Thermo Electron Co., Waltham, MA, USA). Each sample was measured in triplicate. Antioxidant activity was expressed in mg of Trolox/g of sample dry matter.

### 3.9. Mineral Composition

The mineral composition was determined using the ICP–MS method. The ground insects were weighed (0.5 g) into digestion tubes, 10 mL of nitric acid (VWR Chemicals, Darmstadt, Germany), and 2 mL of 30% hydrogen peroxide were added, and predigestion was performed by allowing the samples to stand open for 15 min before sealing. Then, the digestion was performed using a MARS6 microwave digestion system (CEM, Matthews, NC, USA) with the following parameters: microwave power of 1800 kW, ramp time of 20 min, hold time of 15 min, temperature of 210 °C. After that, the samples were cooled down to room temperature, filtered through a 0.22 μm syringe filter (Isolab, Laborgeräte GmbH, Eschau, Germany), and diluted with distilled water. The obtained aliquots were subjected to ICP–MS analysis using an Agilent 7850 ICP mass spectrometer (Agilent Technologies, Santa Clara, CA, USA). High-purity argon as a plasma gas (15.0 L/min), nebulizer gas (1.08 L/min), and auxiliary gas (0.90 L/min) were used. The mineral concentration of the tested material was compared with the certified ICP–MS Calibration Standard (UNSPSC Code 41116107, Agilent Technologies, Santa Clara, CA, USA).

### 3.10. FTIR Measurement

FTIR spectra were investigated using a Spectrum Two UATR Two spectrometer (PerkinElmer, Waltham, USA) equipped with a Universal Attenuated Total Reflectance Accessory (UATR). The scanning was performed in the spectral range of 400–4000 cm^−1^ with a resolution of 4 cm^−1^, and 20 scans of the spectrum were performed. The sample was placed onto the crystal and firmly pressed by a pressure clamp. The analytical data were captured and managed through Spectrum 10 software (PerkinElmer, Waltham, MA, USA).

### 3.11. Allergen Content

Crustacean and mollusk tropomyosin contents were determined using the ELISA method (Demeditec Diagnostics GmbH, Kiel, Germany). The ground insects (1 g) were dissolved in 20 mL of extraction buffer, incubated (15 min at 40 °C), centrifuged (10 min at 2000× *g*), and filtrated. Then, 100 μL of the filtrate was added to the wells according to the manufacturer’s instructions. The absorbance of the final reaction mixture was measured using a microplate reader, BioTek™ 800TS (BioTek, Winooski, VT, USA) at a wavelength of 450 nm (reference wavelength 620 nm) and using a calibration curve prepared for crustacean (0–400 ppb) and mollusk (0–400 ppb) tropomyosin [57]. The analysis was performed in triplicate for each sample.

### 3.12. Microorganism Determination

The ground insects (10 g) were mixed with 90 mL 0.85% NaCl and then homogenized (Stomacher 400 Circulator, Seward, UK) for 5 min. Total viable count was enumerated on plate count agar (PCA) incubated at 30 °C for 72 h. Yeasts and molds were counted on Dichloran Glicerol DG 18 (DG18) agar after incubation at 25 °C for 5 days. *E. coli*, *L. monocytogenes,* and *S. aureus* were counted on chromogenic agar (accordingly, TBX, ALOA, Baird-Parker) after incubation at 37–44 °C for 24–48 h (in accordance with standards). Bacterial endospores were enumerated by heat-shocking insect dilution for 20 min at 80 °C in a sterile tube, after which, pour plates with agar counts (PCA for aerobic or Wilson Blair agar for anaerobic spore-forming bacteria) were incubated at 30 °C for 48 h [57,88]. The number of microorganisms was counted using a ProtoCOL 3 (Synbiosis, Cambridge, UK) and determined in log CFU/g. The presence of *Salmonella* was checked in accordance with the Polish Standard, pre-propagation took place in RVS medium, and then samples were transferred to XLD and Hectoen selective media. All determinations were conducted in triplicate. All media were purchased from Biomaxima, Poland.

### 3.13. Statistical Analysis

The one-way ANOVA procedure with the significance level set at α = 0.05 and the post hoc Tukey’s HSD test were applied to assess the significant differences between the investigated samples using STATISTICA 13.3 (TIBCO Software, Palo Alto, CA, USA).

## 4. Conclusions

The objective of this study was to investigate the impacts of convective drying (CD) at different temperatures (70 and 90 °C) and freeze-drying (FD) on the water activity, color, chemical composition, amino acid profile, oil properties, total polyphenol content, antioxidant properties, mineral composition, allergen content, and microbiological quality of yellow mealworm larvae.

The study demonstrated that the dried insects met the microbiological requirements and exhibited a comparable content of fat (35.1–36.6%) and ash (3.3–3.4%). The freeze-dried insects were characterized by lower water activity and contents of protein, polyphenols, magnesium, iron, and mollusk allergen, but a higher antioxidant activity compared to convective-dried insects. Additionally, freeze-dried insects exhibited lower contents of both essential and non-essential amino acids than convective-dried insects, especially those dried at 90 °C. The oil isolated from freeze-dried insects exhibited significantly higher SFA and MUFA contents and lower oxidative stability, acid, and peroxide values than oil from convective-dried insects.

This study has also demonstrated the influence of the air temperature employed during the CD method. The material dried at a lower temperature (70 °C) showed lower contents of essential and non-essential amino acids, crustacean allergen, and iron, but higher antioxidant activity and mollusk allergen content. Moreover, the contents of SFAs and MUFAs and the peroxide value were found to be lower, while the oxidative stability and acid value were higher.

The results of this study indicate that the freeze-drying method failed to achieve the expected high nutritional quality of dried yellow mealworm larvae. Convective drying, especially at 90 °C, resulted in better quality of dried insects, primarily due to the amino acid profile of the protein. Further analysis should be conducted to assess the bioavailability of amino acids, fatty acids, bioactive compounds, and mineral elements in vitro, focusing on intestinal digestion and absorption.

## Figures and Tables

**Table 1:** Drying time (to moisture ratio MR = 0.02), water activity, and color parameters (L*, a*, b*, BI) of blanched yellow mealworm larvae dried by means of the convective method at 70 °C (Tm_70CD) and 90 °C (Tm_90CD) and the freeze-drying (Tm_FD) method.

	Tm_70CD	Tm_90CD	Tm_FD
Drying Time to MR = 0.02 (min)	175 ± 7 b ^1^	120 ± 7 a	2235 ± 21 c
Water Activity (-)	0.225 ± 0.005 c	0.220 ± 0.008 b	0.068 ± 0.002 a
L*	26.5 ± 0.5 a	34.6 ± 0.7 b	43.7 ± 0.7 c
a*	4.9 ± 0.1 a	6.6 ± 0.1 b	8.3 ± 0.2 c
b*	11.7 ± 0.4 a	16.9 ± 0.3 b	23.6 ± 0.5 c
Browning Index (BI)	70.5 ± 4.5 a	79.7 ± 3.6 b	88.8 ± 4.5 c
Photo	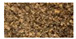	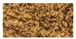	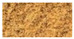

^1^ Different letters within the same row indicate a significant difference between samples (Tukey’s HSD test, *p* < 0.05).

**Table 2:** Chemical composition of blanched yellow mealworm larvae dried by means of the convective method at 70 °C (Tm_70CD) and 90 °C (Tm_90CD) and the freeze-drying (Tm_FD) method.

Component	Tm_70CD	Tm_90CD	Tm_FD
Moisture (%)	3.10 ± 0.05 b ^1^	3.24 ± 0.02 b	1.57 ± 0.31 a
Protein (g/100 g d.m.)	41.14 ± 0.18 b	42.14 ± 0.80 b	37.57 ± 0.05 a
Fat (g/100 g d.m.)	36.62 ± 1.06 a	35.65 ± 1.14 a	35.12 ± 0.30 a
Ash (g/100 g d.m.)	3.42 ± 0.04 a	3.39 ± 0.02 a	3.34 ± 0.04 a

^1^ Different letters within the same row indicate a significant difference between samples (Tukey’s HSD test, *p* < 0.05).

**Table 3:** Amino acid profile of blanched yellow mealworm larvae dried by means of the convective method at 70 °C (Tm_70CD) and 90 °C (Tm_90CD) and the freeze-drying (Tm_FD) method.

Amino Acid (mg/g of Protein)	Tm_70CD	Tm_90CD	Tm_FD
Essential amino acids (EAAs)			
Phenylalanine (Phe)	35.40 ± 0.83 a ^1^	41.60 ± 3.41 b	37.10 ± 2.86 ab
Histidine (His)	42.87 ± 2.50 a	45.44 ± 2.25 a	43.44 ± 4.26 a
Isoleucine (Ile)	36.62 ± 2.02 a	39.89 ± 0.42 a	39.74 ± 5.34 a
Leucine (Leu)	69.42 ± 2.28 ab	75.13 ± 1.10 b	64.40 ± 5.85 a
Lysine (Lys)	54.29 ± 2.12 a	60.73 ± 2.30 a	59.72 ± 6.86 a
Valine (Val)	52.88 ± 1.82 a	58.72 ± 1.57 b	54.14 ± 3.75 ab
Methionine (Met)	4.74 ± 1.38 a	5.78 ± 0.47 a	5.13 ± 1.03 a
Threonine (Thr)	38.68 ± 1.26 a	42.43 ± 0.54 a	41.44 ± 3.53 a
Total EAAs	334.90 ± 10.14 a	369.71 ± 9.08 b	345.10 ± 24.23 ab
Non-essential amino acids (NEAAs)			
Asparagine (Asp)	81.59 ± 3.65 a	86.51 ± 3.94 a	83.92 ± 3.94 a
Serine (Ser)	47.27 ± 0.29 a	51.01 ± 2.24 b	49.11 ± 2.16 ab
Glutamic acid (Glu)	113.81 ± 5.02 a	115.09 ± 5.81 a	111.08 ± 4.07 a
Proline (Pro)	78.96 ± 2.24 a	88.71 ± 1.60 b	80.60 ± 5.50 a
Glycine (Gly)	44.95 ± 1.07 a	49.43 ± 1.96 b	47.74 ± 2.90 ab
Alanine (Ala)	70.14 ± 2.90 ab	76.27 ± 3.08 b	67.69 ± 4.28 a
Cysteine (Cys)	5.97 ± 1.65 a	6.13 ± 0.62 a	4.14 ± 0.91 a
Tyrosine (Tyr)	1.93 ± 0.72 a	5.35 ± 2.37 b	4.80 ± 0.41 b
Arginine (Arg)	49.92 ± 0.92 a	56.44 ± 3.86 b	55.01 ± 1.48 b
Total NEAAs	494.53 ± 12.69 a	534.95 ± 8.26 b	504.09 ± 10.61 a

^1^ Different letters within the same row indicate a significant difference between samples (Tukey’s HSD, *p* < 0.05).

**Table 4:** Fatty acid composition and health indices of oil extracted from blanched yellow mealworm larvae dried by means of the convective method at 70 °C (Tm_70CD) and 90 °C (Tm_90CD) and the freeze-drying (Tm_FD) method.

Fatty Acid (%)	Tm_70CD	Tm_90CD	Tm_FD
Caprylic acid (C8:0)	0.08 ± 0.00 b ^1^	nd	0.02 ± 0.00 a
Capric acid (C10:0)	0.08 ± 0.00 b	0.02 ± 0.00 a	0.02 ± 0.00 a
Lauric acid (C12:0)	0.77 ± 0.00 b	0.59 ± 0.03 a	0.52 ± 0.10 a
Tridecanoic acid (C13:0)	0.05 ± 0.01 a	0.09 ± 0.00 b	0.07 ± 0.02 a
Myristic acid (C14:0)	3.60 ± 0.08 a	4.82 ± 0.14 a	4.38 ± 1.27 a
Pentadecanoic acid (C15:0)	0.07 ± 0.00 a	0.09 ± 0.00 b	0.08 ± 0.01 ab
Palmitic acid (C16:0)	13.48 ± 0.34 a	15.06 ± 0.08 b	15.36 ± 1.25 b
Heptadecanoic acid (C17:0)	0.25 ± 0.03 a	0.29 ± 0.00 b	0.23 ± 0.01 a
Stearic acid (C18:0)	3.08 ± 0.00 a	3.04 ± 0.03 a	3.42 ± 0.16 b
Arachidic acid (C20:0)	0.10 ± 0.02 a	0.10 ± 0.00 a	0.12 ± 0.04 a
Total SFAs	21.55 ± 0.42 a	24.10 ± 0.11 ab	24.21 ± 2.47 b
Myristoleic acid (C14:1)	0.18 ± 0.00 a	0.25 ± 0.01 b	0.20 ± 0.06 ab
Palmitoleic acid (C16:1)	2.19 ± 0.01 a	2.31 ± 0.03 a	2.20 ± 0.18 a
cis-10-Heptadecenoic acid (C17:1)	0.12 ± 0.01 ab	0.12 ± 0.00 b	0.11 ± 0.00 a
Oleic acid (C18:1 n-9c)	52.27 ± 0.50 ab	49.91 ± 0.13 a	53.42 ± 2.38 b
cis-11-Eicosenoic acid (C20:1 n-9c)	0.14 ± 0.01 ab	0.16 ± 0.01 b	0.13 ± 0.01 a
Total MUFAs	54.89 ± 0.52 ab	52.74 ± 0.15 a	56.05 ± 2.14 b
Linoleic acid (C18:2 n-6c)	21.15 ± 0.02 b	20.68 ± 0.02 b	17.52 ± 0.58 a
α-Linolenic acid (C18:3 n-3)	0.83 ± 0.01 b	0.86 ± 0.01 c	0.63 ± 0.01 a
Total PUFAs	21.97 ± 0.03 b	21.53 ± 0.01 b	18.15 ± 0.59 a
Total unidentified fatty acids	1.57 ± 0.16 a	1.64 ± 0.05 a	1.60 ± 0.26 a
Health Indices			
N-6/n-3	25.63 ± 0.19 b	24.18 ± 0.22 a	27.81 ± 0.30 c
Atherogenicity index (AI)	0.37 ± 0.01 a	0.47 ± 0.01 a	0.45 ± 0.10 a
Thrombogenicity index (TI)	0.50 ± 0.01 a	0.58 ± 0.00 ab	0.60 ± 0.08 b
Hypercholesterolemic ratio (HH)	4.35 ± 0.14 b	3.59 ± 0.02 a	3.67 ± 0.62 a

nd—not detected. ^1^ Different letters within the same row indicate a significant difference between samples (Tukey’s HSD, *p* < 0.05).

**Table 5:** Fatty acid composition in the *sn*-1,3 and *sn*-2 positions of TAG and fatty acid percentage share in the *sn*-2 position of TAG (*sn*-2*) of the oil extracted from blanched yellow mealworm larvae dried by means of the convective method at 70 °C (Tm_70CD) and 90 °C (Tm_90CD) and the freeze-drying (Tm_FD) method.

Fatty Acid (%)		Tm_70CD	Tm_90CD	Tm_FD
Myristic acid (C14:0)	*sn*-1,3	4.73 ± 0.02 a ^1^	6.73 ± 0.03 c	6.09 ± 0.04 b
	*sn*-2	1.35 ± 0.04 b	1.00 ± 0.02 a	0.96 ± 0.08 a
	*sn*-2*	12.50 ± 0.39 b	6.88 ± 0.44 a	7.31 ± 0.65 a
Palmitic acid (C16:0)	*sn*-1,3	17.83 ± 0.09 a	21.02 ± 0.01 c	20.46 ± 0.03 b
	*sn*-2	4.79 ± 0.18 b	3.14 ± 0.02 a	5.15 ± 0.06 c
	*sn*-2*	11.83 ± 0.44 c	6.94 ± 0.05 a	11.17 ± 0.14 b
Stearic acid (C18:0)	*sn*-1,3	3.50 ± 0.03 a	3.91 ± 0.02 b	3.92 ± 0.02 b
	*sn*-2	2.24 ± 0.06 b	1.30 ± 0.04 a	2.42 ± 0.05 c
	*sn*-2*	24.24 ± 0.61 b	14.25 ± 0.47 a	23.54 ± 0.48 b
Oleic acid (C18:1 n-9c)	*sn*-1,3	47.47 ± 0.07 b	42.99 ± 0.59 a	47.24 ± 0.76 b
	*sn*-2	61.86 ± 0.13 a	63.74 ± 1.18 a	65.77 ± 1.52 b
	*sn*-2*	39.45 ± 0.09 a	42.57 ± 0.79 c	41.04 ± 0.95 b
Linoleic acid (C18:2 n-6c)	*sn*-1,3	18.75 ± 0.14 c	16.56 ± 0.22 b	14.07 ± 0.08 a
	*sn*-2	25.95 ± 0.29 b	28.91 ± 0.43 c	24.42 ± 0.16 a
	*sn*-2*	40.90 ± 0.46 a	46.60 ± 0.70 b	46.46 ± 0.30 b

^1^ Different letters within the same row indicate a significant difference between samples (Tukey’s HSD, α = 0.05).

**Table 6:** Oxidative stability, acid value, and peroxide value of the oil extracted from blanched yellow mealworm larvae dried by means of the convective method at 70 °C (Tm_70CD) and 90 °C (Tm_90CD) and the freeze-drying (Tm_FD) method.

	Tm_70CD	Tm_90CD	Tm_FD
Oxidative Stability (min)	6.56 ± 0.20 c ^1^	4.76 ± 0.01 b	1.73 ± 0.07 a
Acid Value (mg KOH/g)	16.72 ± 0.94 b	4.65 ± 0.01 a	3.76 ± 0.10 a
Peroxide Value (meq O_2_/kg)	6.55 ± 0.93 b	9.51 ± 1.32 c	<0.01 a

^1^ Different letters within the same row indicate a significant difference between samples (Tukey’s HSD, *p* < 0.05).

**Table 8:** Mineral composition of blanched yellow mealworm larvae dried by means of the convective method at 70 °C (Tm_70CD) and 90 °C (Tm_90CD) and the freeze-drying (Tm_FD) method.

Mineral (mg/100 g d.m.)	Tm_70CD	Tm_90CD	Tm_FD	PRI/AI (mg/day)	%PRI/AI
Potassium (K)	1317.25 ± 12.40 b	1334.22 ± 33.92 b	1173.52 ± 14.40 a	3500	33.53–38.12
Magnesium (Mg)	305.43 ± 7.36 b	291.75 ± 7.44 b	260.79 ± 5.12 a	300	86.93–101.81
Sodium (Na)	169.72 ± 2.13 b ^1^	174.25 ± 2.08 b	153.26 ± 3.01 a	1500	10.22–11.62
Calcium (Ca)	73.16 ± 9.10 a	69.13 ± 12.36 a	65.45 ± 11.09 a	950	6.89–7.70
Zinc (Zn)	18.99 ± 0.40 b	20.04 ± 0.56 b	17.16 ± 0.34 a	9.3	184.56–215.51
Iron (Fe)	5.76 ± 0.13 b	6.31 ± 0.05 c	4.94 ± 0.03 a	11	44.92–57.33
Copper (Cu)	2.60 ± 0.06 b	2.66 ± 0.05 b	2.32 ± 0.04 a	1.3	178.58–204.54
Manganese (Mn)	1.35 ± 0.04 b	1.35 ± 0.02 b	1.19 ± 0.04 a	3	39.58–45.13
Selenium (Se)	0.28 ± 0.00 a	0.27 ± 0.00 a	0.28 ± 0.00 a	0.07	385.29–397.48
Molybdenum (Mo)	0.10 ± 0.00 a	0.10 ± 0.00 a	0.09 ± 0.00 a	0.65	14.45–15.62
Aluminum (Al)	0.26 ± 0.01 b	0.21 ± 0.01 a	0.31 ± 0.00 c	–	–
Barium (Ba)	0.14 ± 0.00 b	0.15 ± 0.00 c	0.12 ± 0.00 a	–	–
Boron (B)	0.10 ± 0.00 b	0.09 ± 0.00 b	0.08 ± 0.01 a	–	–
Chromium (Cr)	0.04 ± 0.00 b	0.04 ± 0.00 a	0.05 ± 0.00 c	–	–
Nickel (Ni)	0.02 ± 0.00 a	0.03 ± 0.00 b	0.03 ± 0.00 ab	–	–
Cobalt (Co)	0.00 ± 0.00 b	0.01 ± 0.00 a	0.01 ± 0.00 a	–	–
Arsenic (As)	1.08 ± 0.02 b	1.00 ± 0.02 a	0.99 ± 0.02 a	–	–
Cadmium (Cd)	0.01 ± 0.00 b	0.01 ± 0.00 c	0.01 ± 0.00 a	–	–

PRI—population reference intake; AI—adequate intake. ^1^ Different letters within the same row indicate a significant difference between samples (Tukey’s HSD, *p* < 0.05).

**Table 9:** Allergen content in blanched yellow mealworm larvae dried by means of the convective method at 70 °C (Tm_70CD) and 90 °C (Tm_90CD) and the freeze-drying (Tm_FD) method.

Allergen (ppb)	Tm_70CD	Tm_90CD	Tm_FD
Crustaceans	4047.24 ± 19.80 a ^1^	4818.91 ± 48.54 b	5274.72 ± 29.83 c
Mollusks	2512.49 ± 32.02 c	2335.48 ± 42.83 b	1952.59 ± 64.31 a

^1^ Different letters within the same row indicate a significant difference between samples (Tukey’s HSD, *p* < 0.05).

**Table 10:** Microbiological quality of blanched yellow mealworm larvae dried by means of the convective method at 70 °C (Tm_70CD) and 90 °C (Tm_90CD) and the freeze-drying (Tm_FD) method.

Microorganism (log CFU/g)	Tm_70CD	Tm_90CD	Tm_FD
Total viable count	2.01 ± 0.12	2.21 ± 0.16	2.10 ± 0.06
Total yeast and mold count	2.29 ± 0.08	1.49 ± 0.16	1.86 ± 0.07
*Listeria monocytogenes*	≤1.00	≤1.00	≤1.00
*Enterobacteriaceae*	≤1.00	≤1.00	≤1.00
*Escherichia coli*	≤1.00	≤1.00	≤1.00
*Staphylococcus aureus*	≤1.00	≤1.00	≤1.00
Aerobic spore-forming bacteria	1.70 ± 0.16	1.56 ± 0.25	1.74 ± 0.10
Anaerobic spore-forming bacteria	≤1.00	≤1.00	≤1.00
Presence of *Salmonella*	absence in 25 g	absence in 25 g	absence in 25 g

## Data Availability

The original contributions presented in the study are included in the article (and supplementary material), further inquiries can be directed to the corresponding authors.

## Supplementary Materials

The following supporting information can be downloaded at https://www.mdpi.com/article/10.3390/molecules29153679/s1, Figure S1: Drying kinetics of blanched and (**a**) convective and (**b**) freeze-dried yellow mealworm larvae.
