# Supplementary material for: The Impact of Drying Methods on the Quality of Blanched Yellow Mealworm (Tenebrio molitor L.) Larvae
Source: Molecules. 2024 Aug 3;29(15):3679. doi: 10.3390/molecules29153679 (PMC11314216; doi:10.3390/molecules29153679)
Supplement: Supplementary file 1 [file molecules-29-03679-s001.zip › molecules-3110117-supplementary.pdf]

Supplementary materials:

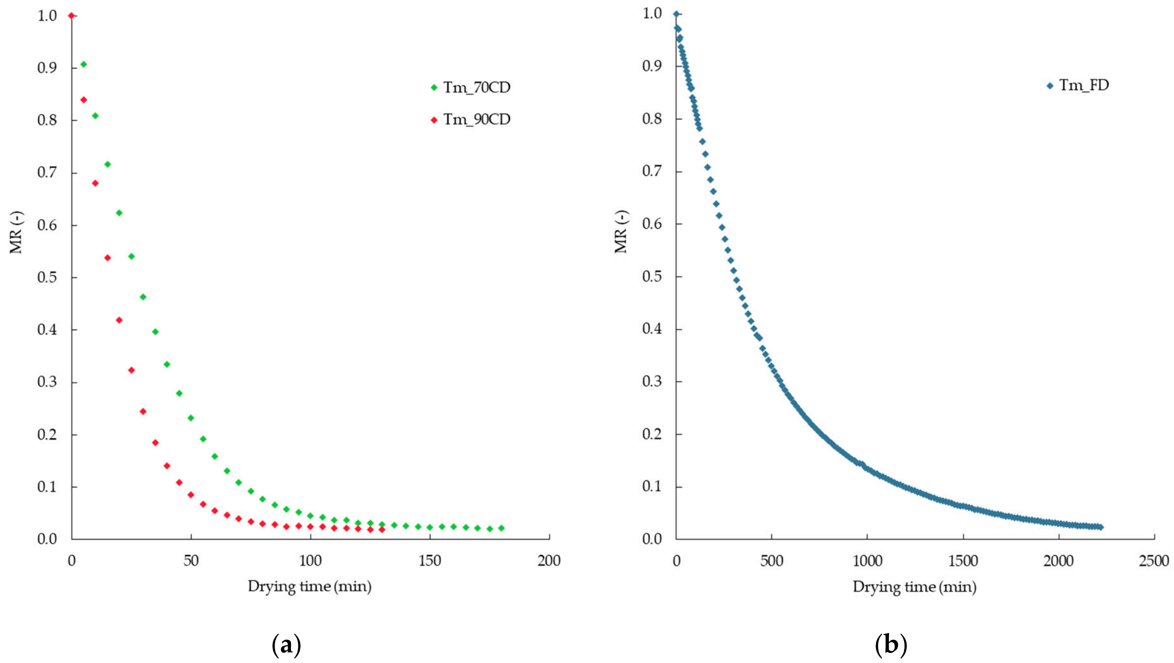

Figure S1. Drying kinetics of blanched and (**a**) convective and (**b**) freeze dried yellow mealworm larvae.
